# Validation of a Scale to Measure Barriers to Promoting Walking in Hospitalized Older Adults

**DOI:** 10.1093/geroni/igaf122.3368

**Published:** 2025-12-31

**Authors:** Carolanne Bianchi, Tobie Olsan, Joyce Smith

**Affiliations:** University of Rochester School of Nursing, Rochester, New York, United States; University of Rochester, Rochester, New York, United States; University of Rochester, Rochester, New York, United States

## Abstract

This study validated the Barriers to Promoting Walking Scale (BPWS) to measure interprofessional team members’ perceptions of barriers to walking hospitalized older adults. Addressing the barriers to closing the knowledge-practice gap between promoting walking and the high prevalence of low mobility during hospitalization is critical to improving the effectiveness of quality improvement interventions and reducing hospital-associated functional decline. To assess the psychometric properties of the BPWS, the instrument was administered to two purposive samples of interprofessional team members from medical, surgical, and combined medical-surgical units in two hospitals resulting in 239/726 completed surveys (33% response rate). Descriptive statistics were used to examine distributional characteristics of the variables. Internal reliability was calculated using Cronbach α. Construct validity was evaluated by measuring the correlation of each domain score with the entire scale score and by known-group validity. Rasch analysis was used to eliminate ambiguous scale items or those with low variability. Scale items—developed from published literature, focus groups, and expert review demonstrated face and content validity (S-CVI/Ave, 0.91). Exploratory factor analysis produced a three-factor solution (Team Members’ Confidence in Walking Patients, Practices That Limit Patient Walking, and Patient and Family Engagement) with adequate reliability (Cronbach α = 0.75 to 0.91) and construct validity. This study provides strong evidence of the reliability and validity of the BPWS. Engaging the interdisciplinary team and identifying their perceived barriers to walking hospitalized older adults provides opportunities to create shared understandings of older adult care and design novel solutions to promote walking and mitigate hospital acquired functional decline.

